# Ventricular Tachycardia Precipitated by Short-long-short Sequence in a Patient with Implantable-cardioverter Defibrillator

**DOI:** 10.1016/s0972-6292(16)30461-2

**Published:** 2012-01-31

**Authors:** Diego Chemello, Anandaraja Subramanian, Sheila Watkins, Douglas Cameron

**Affiliations:** Toronto General Hospital, University Health Network, Toronto, Canada

**Keywords:** Ventricular Tachycardia, Short-long-short Sequence, Implantable-cardioverter Defibrillator

## Abstract

Abrupt changes in heart rate, particularly short-long-short sequences in the ventricular cycle length (CL), might precede initiation of ventricular tachycardia/fibrillation (VT/VF). These changes may be facilitated or caused by pacing activity in patients with pacemakers or implantable-cardioverter defibrillators (ICDs). We describe a patient with two episodes of acquired VT precipitated by short-long-short sequences and diagnosed from the ICD recordings. In such cases, the knowledge of the device parameters is extremely important for a correct diagnosis and management.

## Case Report

A 59-years-old man with nonischemic dilated cardiomyopathy was admitted to the Emergency Department after receiving a shock from his ICD, which was preceded by dizziness. The dual-chamber ICD (Medtronic EnTrust, Minneapolis, MN, USA) was implanted two years earlier for primary prevention of sudden cardiac death. At the time of device implantation, patient was in sinus rhythm with normal PR interval (170 ms) and therefore it was programmed to AAI+ mode (MVP™) at 60 bpm. Therapies were programmed as follows: 1) VT zone at 176 bpm: anti-tachycardia pacing (ATP) (Burst x3, Ramp x2), followed by shock (30J, 35J x3); 2) VF zone at > 200 bpm: shock (30J, 35J, 35J x4) with ATP During Charging™.

Device interrogation currently demonstrated normal function. Two episodes of ventricular arrhythmias were logged by the device, both of them in the VF zone. The first one terminated spontaneously and no therapies were delivered. The second episode occurred the following day and was terminated after a shock. In the present case, both episodes can be explained by the same mechanism.

**Figure 1:**
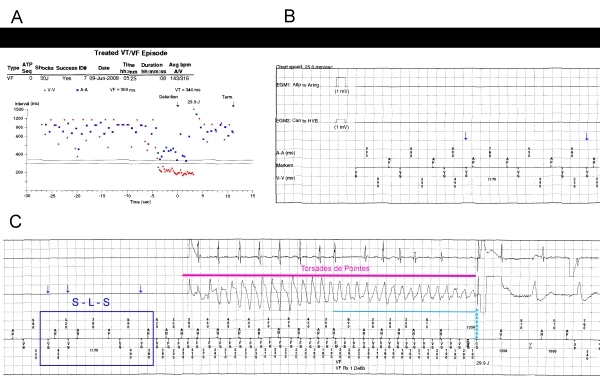
Dot plot (A) and electrograms (B and C) during one episode of ventricular tachycardia. An abrupt increase in the ventricular cycle length with atrio-ventricular dissociation is observed. In C, another short-long-short sequence is highlighted (blue square), followed by ventricular tachycardia. After detection of the episode, device started to charge (blue line); the shock was delivered, which terminated the tachycardia (arrows head). The blue arrows in B and C represent premature ventricular complexes.

Figure 1 shows the dot plot and cardiac electrograms (EGMs) during the episode treated with shock. In the beginning, patient rhythm was atrial pacing (AP) with intrinsic atrioventricular (AV) conduction and ventricular sensed events (VS). However, some VS events occurred before AP, indicating premature ventricular complexes (PVCs). Suddenly, there is an increase in the ventricular rate to a CL of 170-240ms, which fell into the VF detection zone and was successfully treated with a 30J shock. The EGM before the tachycardia episode shows a device pacing initially in AAI mode. This is inferred form the fact that the AV interval is longer than the programmed sensed AV interval (160ms). The first AP beat is followed by a VS event, representing normal AV conduction. The second and third beats are PVCs with retrograde atrial conduction (AR). According to the MVP algorithm, after a PVC, AP is inhibited and a new ventriculo-atrial escape interval is started. After these PVCs, the absence of intrinsic atrial activity resulted in an AP beat. The sequence of couplets of PVCs, separated by a long pause was the responsible for a short-long-short (S-L-S) sequence, which triggered VT.

## Discussion

The present case illustrates the importance of S-L-S sequences as a trigger for ventricular arrhythmias. Despite not frequently seen in clinical practice, this mechanism had been previously described as a potential source of VT initiation, either spontaneously or induced by paced beats in patients with ICD [1,2]. This mechanism of initiation does not depend of the heart disease etiology [3]. A recent analysis of 1,356 VT/VF episodes in patients with ICDs revealed that S-L-S sequence can be responsible for up to 30% of cases of VT/VF, independent of the pacing mode [4]. Some pacing modes characteristically facilitate bradycardia and can be a potential source of pause-dependent VT/VF. Overdrive pacing and dedicated pause suppression techniques might be useful in selected patients, but not for all. In one small study, pacing-facilitated VT/VF was decreased by pacing deactivation, despite increased duration of pauses. This last observation supports the fact that not only the CL variations, but also the pacing stimulus, induce repolarization abnormalities with potential proarrhythmic effects [5]. Though the MVP mode has been shown to predispose to long short sequences and VT in some patient with AV block and frequent VPCs, the mechanism in the present case was independent of the MVP mode and device settings were not changed. The patient was put on a higher dose of beta-blockers with significant suppression of PVCs and no further shocks in 12 months.
